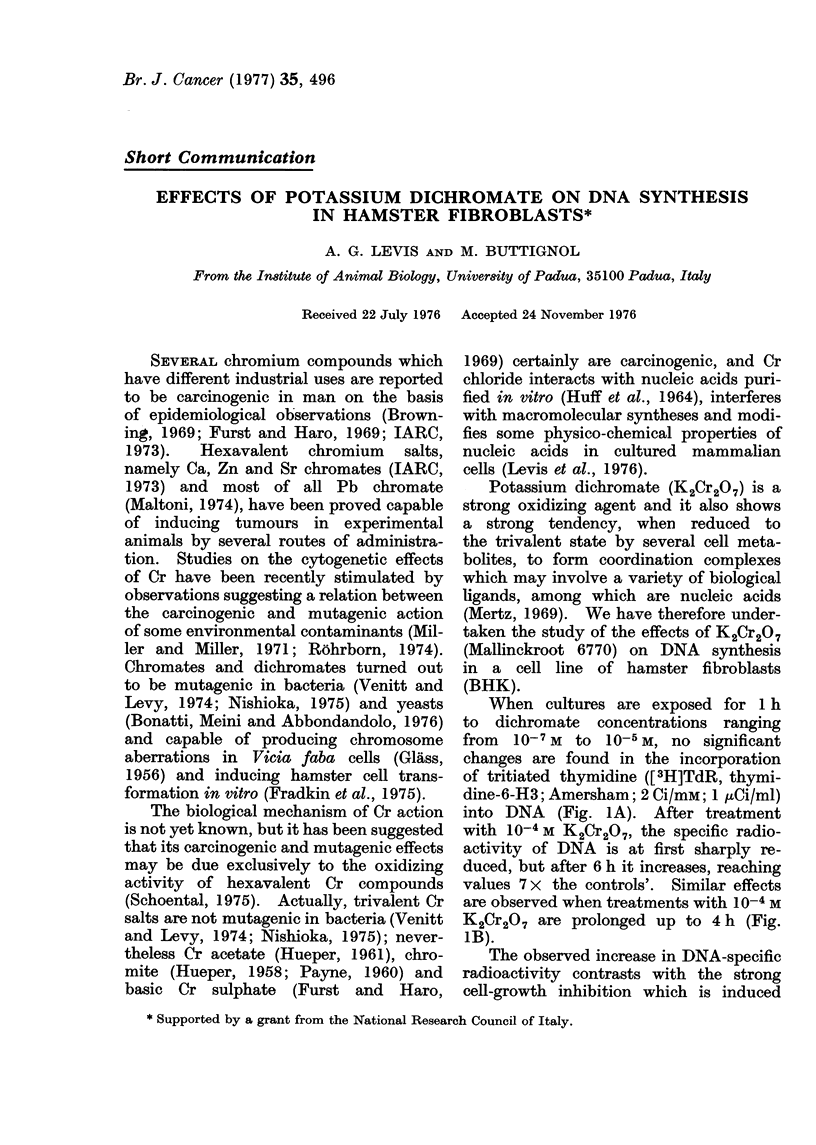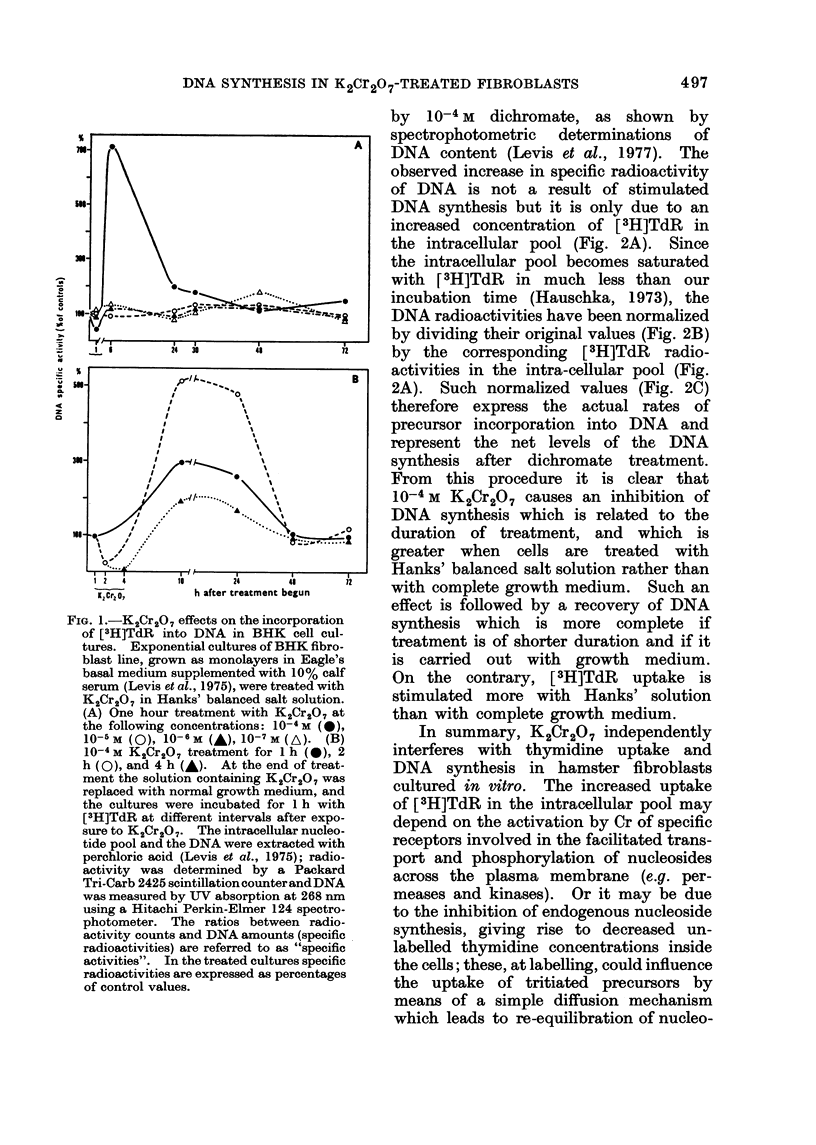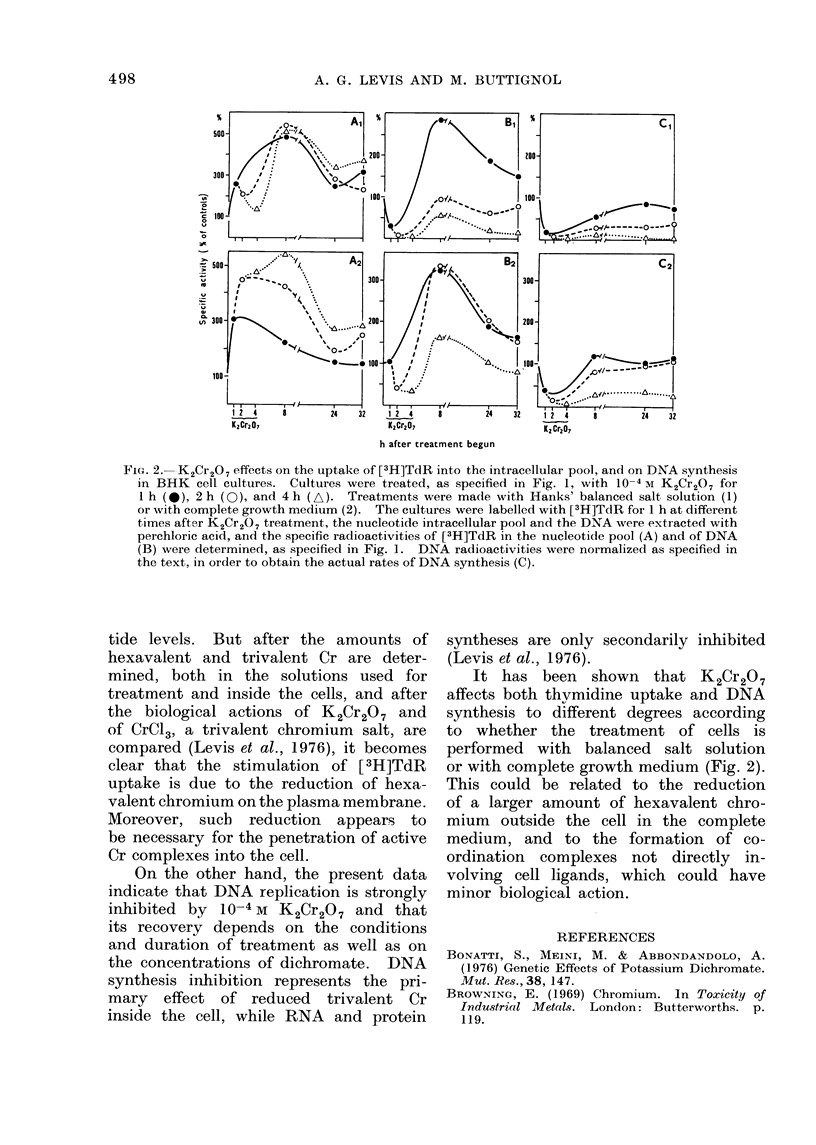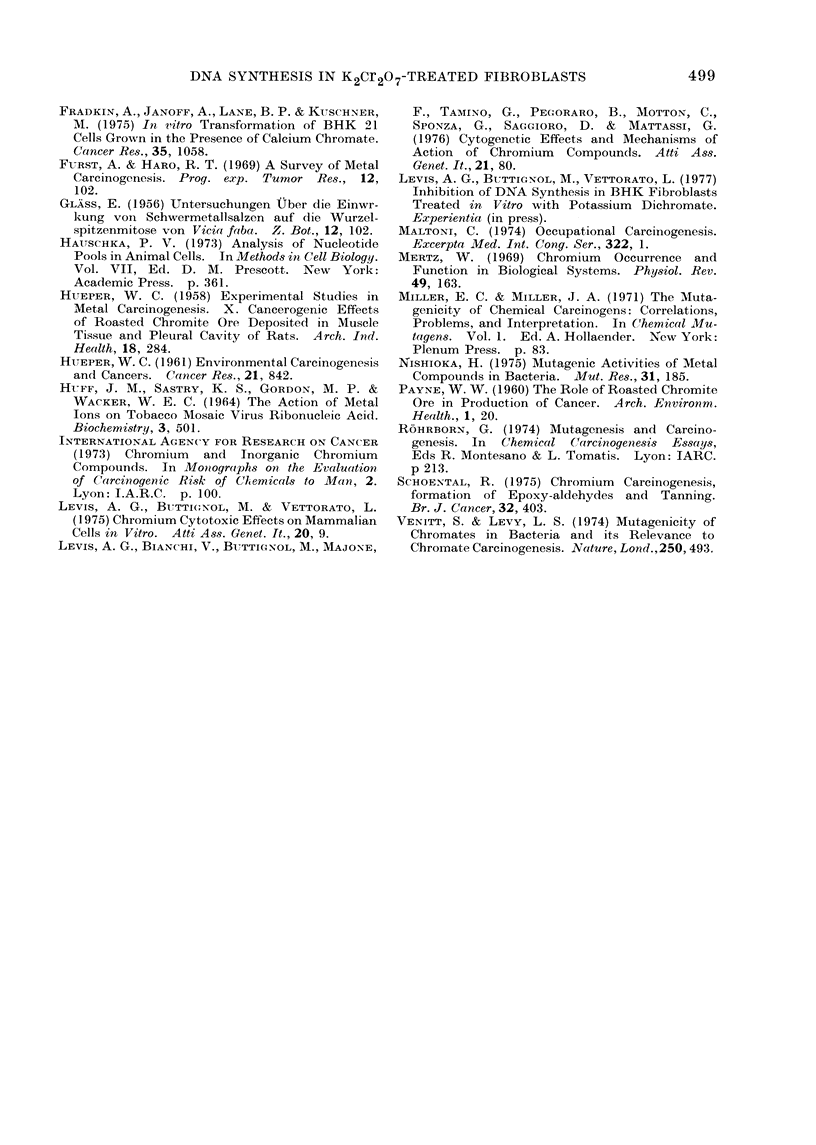# Effects of potassium dichromate on DNA synthesis in hamster fibroblasts.

**DOI:** 10.1038/bjc.1977.75

**Published:** 1977-04

**Authors:** A. G. Levis, M. Buttignol


*Br. J. Cancer* (1977) **35**, 496

***Short Communication***

### EFFECTS OF POTASSIUM DICHROMATE ON DNA SYNTHESIS IN HAMSTER FIBROBLASTS*

A. G. LEVIS AND M. BUTTIGNOL

*From the Institute of Animal Biology, University of Padua, 35100 Padua, Italy*

Received 22 July 1976 Accepted 24 November 1976

SEVERAL chromium compounds which have different industrial uses are reported to be carcinogenic in man on the basis of epidemiological observations (Browning, 1969; Furst and Haro, 1969; IARC, 1973). Hexavalent chromium salts, namely Ca, Zn and Sr chromates (IARC, 1973) and most of all Pb chromate (Maltoni, 1974), have been proved capable of inducing tumours in experimental animals by several routes of administration. Studies on the cytogenetic effects of Cr have been recently stimulated by observations suggesting a relation between the carcinogenic and mutagenic action of some environmental contaminants (Miller and Miller, 1971; Röhrborn, 1974). Chromates and dichromates turned out to be mutagenic in bacteria (Venitt and Levy, 1974; Nishioka, 1975) and yeasts (Bonatti, Meini and Abbondandolo, 1976) and capable of producing chromosome aberrations in *Vicia faba* cells (Gläss, 1956) and inducing hamster cell transformation *in vitro* (Fradkin *et al.*, 1975).

The biological mechanism of Cr action is not yet known, but it has been suggested that its carcinogenic and mutagenic effects may be due exclusively to the oxidizing activity of hexavalent Cr compounds (Schoental, 1975). Actually, trivalent Cr salts are not mutagenic in bacteria (Venitt and Levy, 1974; Nishioka, 1975); nevertheless Cr acetate (Hueper, 1961), chromite (Hueper, 1958; Payne, 1960) and basic Cr sulphate (Furst and Haro, 1969) certainly are carcinogenic, and Cr chloride interacts with nucleic acids purified *in vitro* (Huff *et al.*, 1964), interferes with macromolecular syntheses and modifies some physico-chemical properties of nucleic acids in cultured mammalian cells (Levis *et al.*, 1976).

Potassium dichromate ($K_2Cr_2O_7$) is a strong oxidizing agent and it also shows a strong tendency, when reduced to the trivalent state by several cell metabolites, to form coordination complexes which may involve a variety of biological ligands, among which are nucleic acids (Mertz, 1969). We have therefore undertaken the study of the effects of $K_2Cr_2O_7$ (Mallinckroot 6770) on DNA synthesis in a cell line of hamster fibroblasts (BHK).

When cultures are exposed for 1 h to dichromate concentrations ranging from $10^{-7}$ M to $10^{-5}$ M, no significant changes are found in the incorporation of tritiated thymidine ([$^3$H]TdR, thymidine-6-H3; Amersham; 2 Ci/mM; 1 $\mu$Ci/ml) into DNA (Fig. 1A). After treatment with $10^{-4}$ M $K_2Cr_2O_7$, the specific radioactivity of DNA is at first sharply reduced, but after 6 h it increases, reaching values 7× the controls'. Similar effects are observed when treatments with $10^{-4}$ M $K_2Cr_2O_7$ are prolonged up to 4 h (Fig. 1B).

The observed increase in DNA-specific radioactivity contrasts with the strong cell-growth inhibition which is induced

* Supported by a grant from the National Research Council of Italy.

FIG. 1.—$K_2Cr_2O_7$ effects on the incorporation of [$^3$H]TdR into DNA in BHK cell cultures. Exponential cultures of BHK fibroblast line, grown as monolayers in Eagle's basal medium supplemented with 10% calf serum (Levis *et al.*, 1975), were treated with $K_2Cr_2O_7$ in Hanks' balanced salt solution. (A) One hour treatment with $K_2Cr_2O_7$ at the following concentrations: $10^{-4}$ M (●), $10^{-5}$ M (○), $10^{-6}$ M (▲), $10^{-7}$ M (△). (B) $10^{-4}$ M $K_2Cr_2O_7$ treatment for 1 h (●), 2 h (○), and 4 h (▲). At the end of treatment the solution containing $K_2Cr_2O_7$ was replaced with normal growth medium, and the cultures were incubated for 1 h with [$^3$H]TdR at different intervals after exposure to $K_2Cr_2O_7$. The intracellular nucleotide pool and the DNA were extracted with perchloric acid (Levis *et al.*, 1975); radioactivity was determined by a Packard Tri-Carb 2425 scintillation counter and DNA was measured by UV absorption at 268 nm using a Hitachi Perkin-Elmer 124 spectrophotometer. The ratios between radioactivity counts and DNA amounts (specific radioactivities) are referred to as "specific activities". In the treated cultures specific radioactivities are expressed as percentages of control values.

by $10^{-4}$ M dichromate, as shown by spectrophotometric determinations of DNA content (Levis *et al.*, 1977). The observed increase in specific radioactivity of DNA is not a result of stimulated DNA synthesis but it is only due to an increased concentration of [$^3$H]TdR in the intracellular pool (Fig. 2A). Since the intracellular pool becomes saturated with [$^3$H]TdR in much less than our incubation time (Hauschka, 1973), the DNA radioactivities have been normalized by dividing their original values (Fig. 2B) by the corresponding [$^3$H]TdR radioactivities in the intra-cellular pool (Fig. 2A). Such normalized values (Fig. 2C) therefore express the actual rates of precursor incorporation into DNA and represent the net levels of the DNA synthesis after dichromate treatment. From this procedure it is clear that $10^{-4}$ M $K_2Cr_2O_7$ causes an inhibition of DNA synthesis which is related to the duration of treatment, and which is greater when cells are treated with Hanks' balanced salt solution rather than with complete growth medium. Such an effect is followed by a recovery of DNA synthesis which is more complete if treatment is of shorter duration and if it is carried out with growth medium. On the contrary, [$^3$H]TdR uptake is stimulated more with Hanks' solution than with complete growth medium.

In summary, $K_2Cr_2O_7$ independently interferes with thymidine uptake and DNA synthesis in hamster fibroblasts cultured *in vitro*. The increased uptake of [$^3$H]TdR in the intracellular pool may depend on the activation by Cr of specific receptors involved in the facilitated transport and phosphorylation of nucleosides across the plasma membrane (*e.g.* permeases and kinases). Or it may be due to the inhibition of endogenous nucleoside synthesis, giving rise to decreased unlabelled thymidine concentrations inside the cells; these, at labelling, could influence the uptake of tritiated precursors by means of a simple diffusion mechanism which leads to re-equilibration of nucleo-

FIG. 2.—$K_2Cr_2O_7$ effects on the uptake of [$^3$H]TdR into the intracellular pool, and on DNA synthesis in BHK cell cultures. Cultures were treated, as specified in Fig. 1, with $10^{-4}$ M $K_2Cr_2O_7$ for 1 h (●), 2 h (○), and 4 h (△). Treatments were made with Hanks' balanced salt solution (1) or with complete growth medium (2). The cultures were labelled with [$^3$H]TdR for 1 h at different times after $K_2Cr_2O_7$ treatment, the nucleotide intracellular pool and the DNA were extracted with perchloric acid, and the specific radioactivities of [$^3$H]TdR in the nucleotide pool (A) and of DNA (B) were determined, as specified in Fig. 1. DNA radioactivities were normalized as specified in the text, in order to obtain the actual rates of DNA synthesis (C).

tide levels. But after the amounts of hexavalent and trivalent Cr are determined, both in the solutions used for treatment and inside the cells, and after the biological actions of $K_2Cr_2O_7$ and of $CrCl_3$, a trivalent chromium salt, are compared (Levis *et al.*, 1976), it becomes clear that the stimulation of [$^3$H]TdR uptake is due to the reduction of hexavalent chromium on the plasma membrane. Moreover, such reduction appears to be necessary for the penetration of active Cr complexes into the cell.

On the other hand, the present data indicate that DNA replication is strongly inhibited by $10^{-4}$ M $K_2Cr_2O_7$ and that its recovery depends on the conditions and duration of treatment as well as on the concentrations of dichromate. DNA synthesis inhibition represents the primary effect of reduced trivalent Cr inside the cell, while RNA and protein syntheses are only secondarily inhibited (Levis *et al.*, 1976).

It has been shown that $K_2Cr_2O_7$ affects both thymidine uptake and DNA synthesis to different degrees according to whether the treatment of cells is performed with balanced salt solution or with complete growth medium (Fig. 2). This could be related to the reduction of a larger amount of hexavalent chromium outside the cell in the complete medium, and to the formation of coordination complexes not directly involving cell ligands, which could have minor biological action.

### REFERENCES

BONATTI, S., MEINI, M. & ABBONDANDOLO, A. (1976) Genetic Effects of Potassium Dichromate. *Mut. Res.*, **38**, 147.

BROWNING, E. (1969) Chromium. In *Toxicity of Industrial Metals*. London: Butterworths. p. 119.

FRADKIN, A., JANOFF, A., LANE, B. P. & KUSCHNER, M. (1975) *In vitro* Transformation of BHK 21 Cells Grown in the Presence of Calcium Chromate. *Cancer Res.*, **35**, 1058.

FURST, A. & HARO, R. T. (1969) A Survey of Metal Carcinogenesis. *Prog. exp. Tumor Res.*, **12**, 102.

GLÄSS, E. (1956) Untersuchungen Über die Einwrkung von Schwermetallsalzen auf die Wurzelspitzenmitose von *Vicia faba*. *Z. Bot.*, **12**, 102.

HAUSCHKA, P. V. (1973) Analysis of Nucleotide Pools in Animal Cells. In *Methods in Cell Biology*. Vol. VII, Ed. D. M. Prescott. New York: Academic Press. p. 361.

HUEPER, W. C. (1958) Experimental Studies in Metal Carcinogenesis. X. Cancerogenic Effects of Roasted Chromite Ore Deposited in Muscle Tissue and Pleural Cavity of Rats. *Arch. Ind. Health*, **18**, 284.

HUEPER, W. C. (1961) Environmental Carcinogenesis and Cancers. *Cancer Res.*, **21**, 842.

HUFF, J. M., SASTRY, K. S., GORDON, M. P. & WACKER, W. E. C. (1964) The Action of Metal Ions on Tobacco Mosaic Virus Ribonucleic Acid. *Biochemistry*, **3**, 501.

INTERNATIONAL AGENCY FOR RESEARCH ON CANCER (1973) Chromium and Inorganic Chromium Compounds. In *Monographs on the Evaluation of Carcinogenic Risk of Chemicals to Man*, **2**. Lyon: I.A.R.C. p. 100.

LEVIS, A. G., BUTTIGNOL, M. & VETTORATO, L. (1975) Chromium Cytotoxic Effects on Mammalian Cells *in Vitro*. *Atti Ass. Genet. It.*, **20**, 9.

LEVIS, A. G., BIANCHI, V., BUTTIGNOL, M., MAJONE, F., TAMINO, G., PEGORARO, B., MOTTON, C., SPONZA, G., SAGGIORO, D. & MATTASSI, G. (1976) Cytogenetic Effects and Mechanisms of Action of Chromium Compounds. *Atti Ass. Genet. It.*, **21**, 80.

LEVIS, A. G., BUTTIGNOL, M., VETTORATO, L. (1977) Inhibition of DNA Synthesis in BHK Fibroblasts Treated *in Vitro* with Potassium Dichromate. *Experientia* (in press).

MALTONI, C. (1974) Occupational Carcinogenesis. *Excerpta Med. Int. Cong. Ser.*, **322**, 1.

MERTZ, W. (1969) Chromium Occurrence and Function in Biological Systems. *Physiol. Rev.* **49**, 163.

MILLER, E. C. & MILLER, J. A. (1971) The Mutagenicity of Chemical Carcinogens: Correlations, Problems, and Interpretation. In *Chemical Mutagens*. Vol. 1. Ed. A. Hollaender. New York: Plenum Press. p. 83.

NISHIOKA, H. (1975) Mutagenic Activities of Metal Compounds in Bacteria. *Mut. Res.*, **31**, 185.

PAYNE, W. W. (1960) The Role of Roasted Chromite Ore in Production of Cancer. *Arch. Environm. Health.*, **1**, 20.

RÖHRBORN, G. (1974) Mutagenesis and Carcinogenesis. In *Chemical Carcinogenesis Essays*, Eds R. Montesano & L. Tomatis. Lyon: IARC. p 213.

SCHOENTAL, R. (1975) Chromium Carcinogenesis, formation of Epoxy-aldehydes and Tanning. *Br. J. Cancer*, **32**, 403.

VENITT, S. & LEVY, L. S. (1974) Mutagenicity of Chromates in Bacteria and its Relevance to Chromate Carcinogenesis. *Nature, Lond.*, **250**, 493.